# Supplementary material for: Fabrication and Characterization of Polysaccharide Metallohydrogel Obtained from Succinoglycan and Trivalent Chromium
Source: Polymers (Basel). 2021 Jan 8;13(2):202. doi: 10.3390/polym13020202 (PMC7827257; doi:10.3390/polym13020202)
Supplement: Supplementary file 1 [file polymers-13-00202-s001.pdf]

polymers

MDPI

Supplementary Materials

Fabrication and characterization of polysaccharide metallohydrogel obtained from succinoglycan and trivalent chromium

Dajung Kim [1], Seonmok Kim [1] and Seunho Jung [1,2*]

[1] Department of Bioscience and Biotechnology, Microbial Carbohydrate Resource Bank (MCRB), Konkuk University, Seoul 05029, Korea; dajung903@naver.com (D.K.); gkdurk9999@naver.com (S.K.)
[2] D Department of Systems Biotechnology & Institute for Ubiquitous Information Technology and Applications (UBITA), Center for Biotechnology Research in UBITA (CBRU), Konkuk University, Seoul 05029, Korea
* Correspondence: shjung@konkuk.ac.kr; Tel.:+82-2-450-3520+

Received: date; Accepted: date; Published: date

Table of Contents

1. The molecular weight and GPC measurement of succinoglycan p2

2. Viscosity change during the first heating cycle of succinoglycan p2

3. The pH-dependent gelling effect of an aqueous succinoglycan solution based on Cr^{3+} concentration change p2

4. Reversible gel phase transition of SCx hydrogel triggered by a pH change p3

5. Comparison of mechanical properties of $SC_{26.4}$ after the changes in pH of solutions initially prepared in strong acids (pH 1) and strong bases (pH 9)) p3

6. Swelling ratio curves for SCx in distilled water at 25°C p4

7. UV–vis spectra of Cr^{3+} solution (5mM) and SCx immersed in D.W p4

Table S1. The molecular weight and GPC measurement of succinoglycan.

Sample name	Retention time (min)	% Area	% Height	Mn	Mw	Polydispersity
Succinoglycan	32.371	100.00	100.00	16924	180200	1.064715

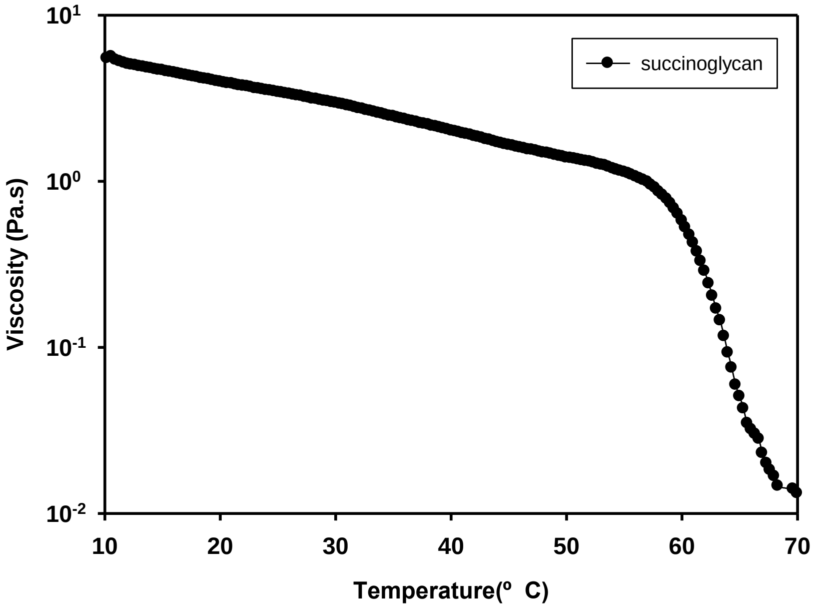

Figure S1. Viscosity change during the first heating cycle of succinoglycan (aq, wt. 1%). Temperature ramp rheological studies were conducted in the range of 10-70 degrees at a constant frequency and strain.

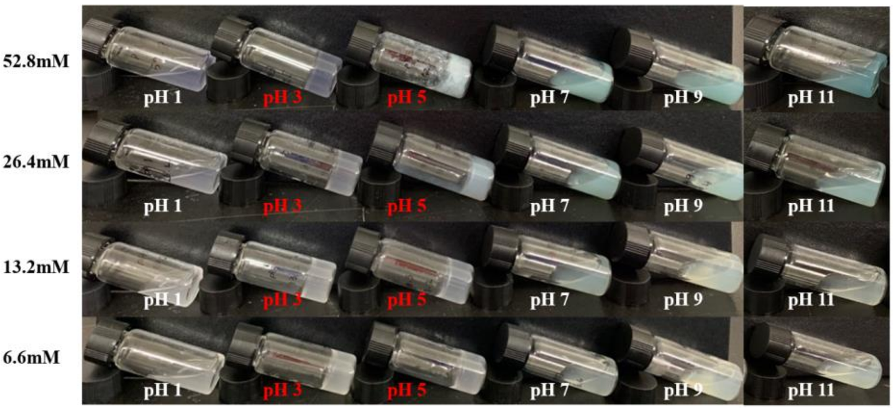

Figure S2. The pH-dependent gelling effect of an aqueous succinoglycan solution based on Cr^{3+} concentration change. The pH was adjusted by adding a small amount of 01. M HCl and 0.1 M NaOH aqueous solution.

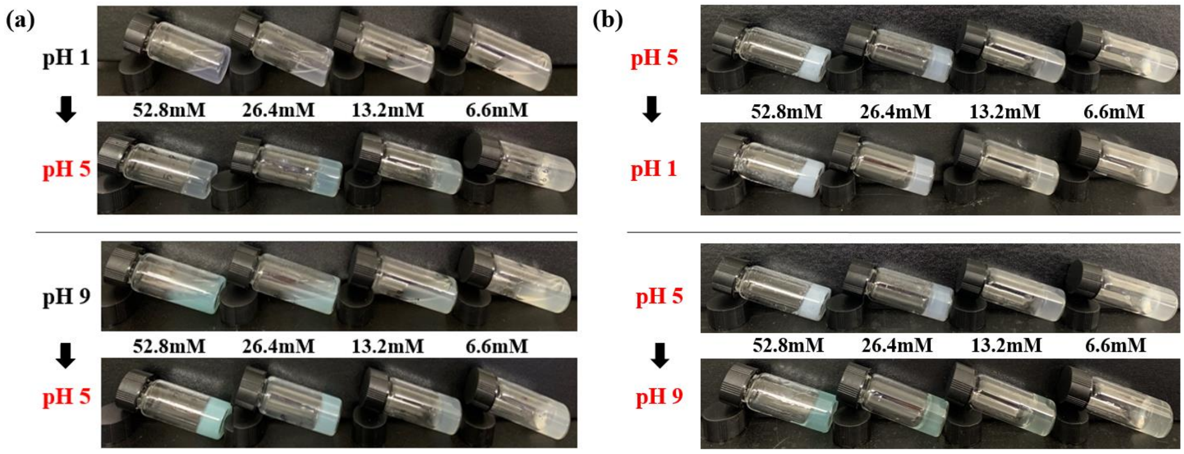

Figure S3. Reversible gel phase transition of SCx triggered by a pH change (a) from 1 to 5 and 9 to 5, (b) from 5 to 1 and 5 to 9.

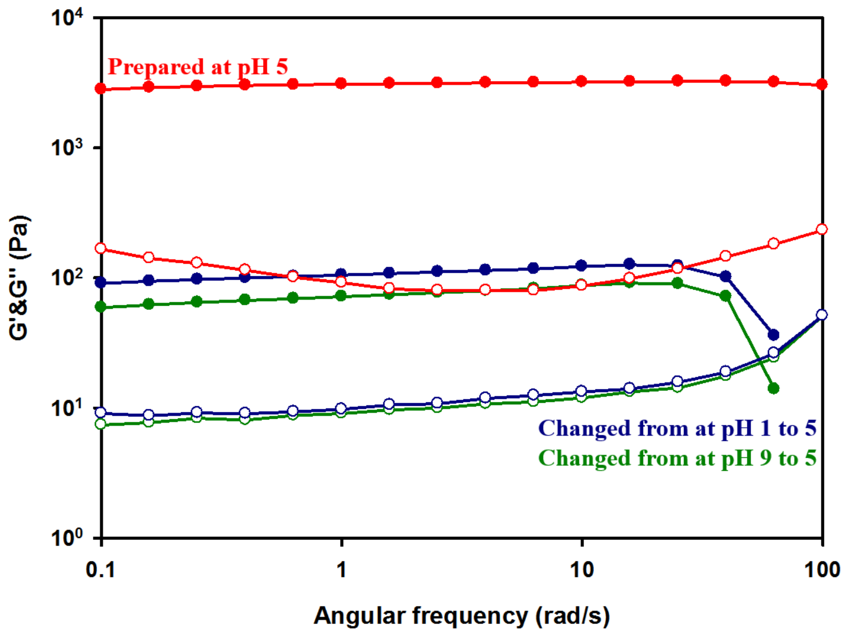

Figure S4. Comparison of mechanical properties of $SC_{26.4}$ after the changes in pH of solutions initially prepared in strong acids (pH 1) and strong bases (pH 9). Storage modulus (G′, filled symbols) and loss modulus (G″, empty symbols) of hydrogels.

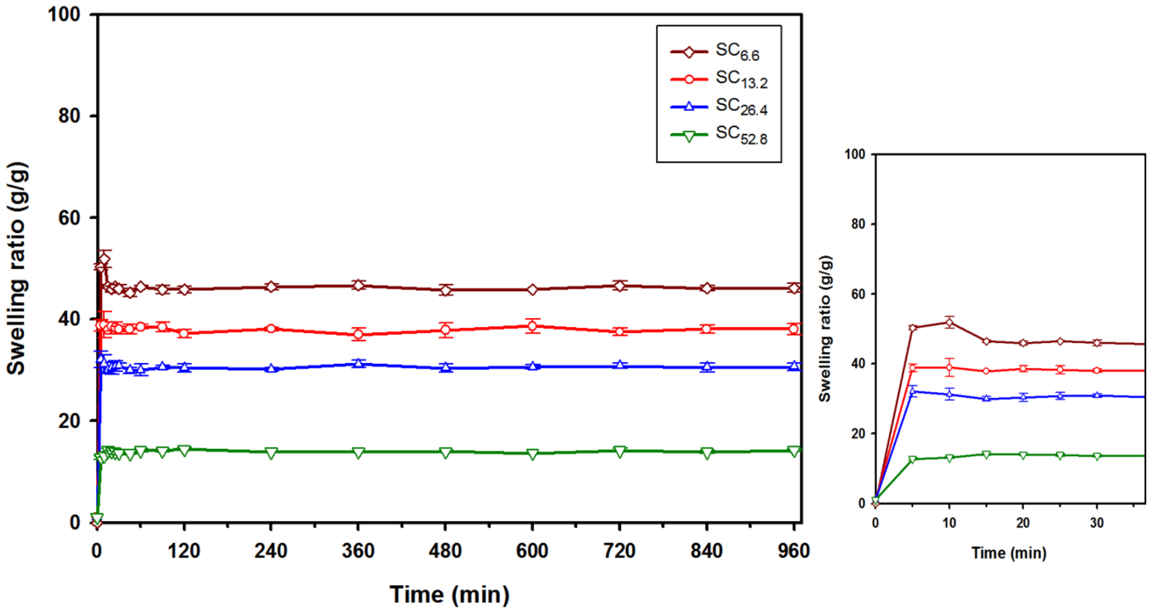

Figure S5. Swelling ratio curves for SCx in distilled water at 25°C.

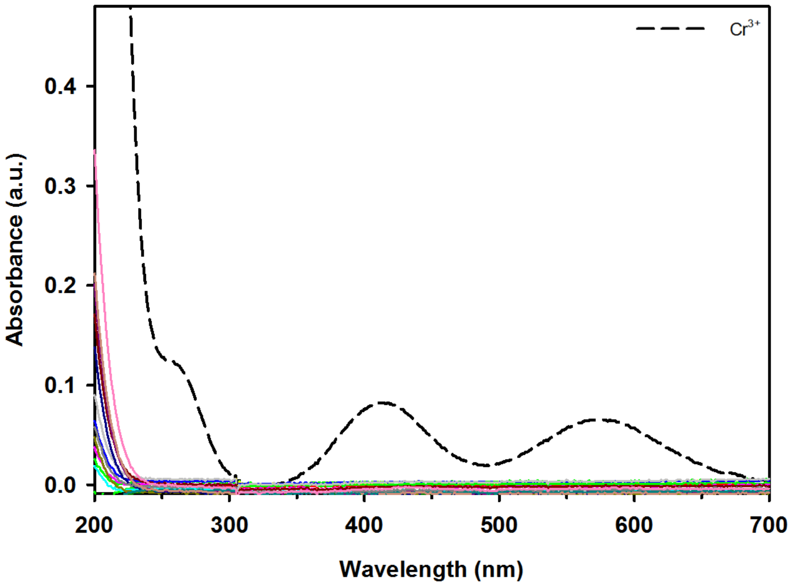

Figure S6. UV–vis spectra of Cr^{3+} solution (5mM) and SCx immersed in D.W.
